# Microwave power sources for industrial, scientific and medical applications

**DOI:** 10.1098/rsta.2024.0069

**Published:** 2025-05-22

**Authors:** Steve Cripps

**Affiliations:** ^1^ Cardiff University, Cardiff, UK

**Keywords:** microwave, power, non-thermal effects

## Abstract

The industrial, scientific and medical (ISM) sector has been a growth area in recent years for applications of microwave engineering. These applications include various forms of heating and more controversial uses that exploit so-called ‘non-thermal’ effects of microwave exposure on biological and chemical samples. Given the non-thermal nature of these effects, the microwave power source may be adequate in pulsed, rather than continuous form. This paper will not attempt to address the questions surrounding the provenance of such effects but will discuss the challenges presented to the microwave circuit designer in delivering substantial amounts of microwave power, both continuous and pulsed, to targets that vary in size and microwave impedance properties. ISM applications may represent a large new market for radio frequency power amplifier (RFPA) products that may utilize alternative technologies and design approaches over those that have evolved for the conventional microwave applications such as telecommunications and radar.

This article is part of the discussion meeting issue ‘Microwave science in sustainability’.

## 1. Introduction

Recent years have seen some important diversification in the applications of microwaves, which for the purposes of this paper, will be grouped under the general heading of industrial, scientific and medical (ISM). These can be summarized as follows:

— Industrial heating (large volumes).
— Domestic heating (microwave cookers).
— Selective heating (small volumes).
— Biological (‘non-thermal’ microwave exposure).
— Medical (various).
— Microwave chemistry.
— Spectroscopy, including electron parametric resonance.

These applications are mostly still emerging and are not treated in any detail in this paper; in particular, non-thermal effects on cells exposed to microwave radiation remain a controversial subject [1]. The focus is on recognizing that these applications currently make use of commercial radio frequency power amplifier (RFPA) products that are designed primarily to address the needs of the communications and military industries, which can be summarized as follows:

— Designed to operate and meet specifications only in a 50 Ohm environment, input and output.
— Designed to operate to full specification over a specified frequency range, both in single and multi-signal environments.
— For RFPAs, linearity and efficiency are usually critical specifications.
— Output power is usually specified as continuous, not pulsed.

ISM applications can have substantially different requirements and characteristics, which can be summarized as follows:

— Target ‘applicator’ is usually nowhere near 50 Ohms (usually some form of lossy resonator).
— Impedance presented to the power amplifier (PA) can be a ‘moving goalpost’, both during exposure and for different material samples and emplacements.
— Linearity usually unimportant (e.g. PA can be well saturated).
— Efficiency may be important in portable applications.
— Spectral purity not critical; PAs can be turned into oscillators.

## 2. The 50 Ohm interface

**Figure 1.**
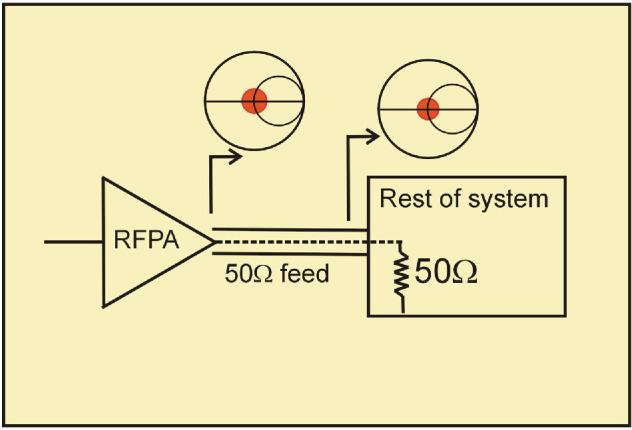
50 Ohm microwave system.

A conventional microwave system design requires a standardized impedance environment:

As shown in figure 1, if every system component is designed to present and operate with 50 Ohm terminations, the various interconnections can be performed using essentially ‘random’ lengths of 50 Ohm transmission line.

**Figure 2.**
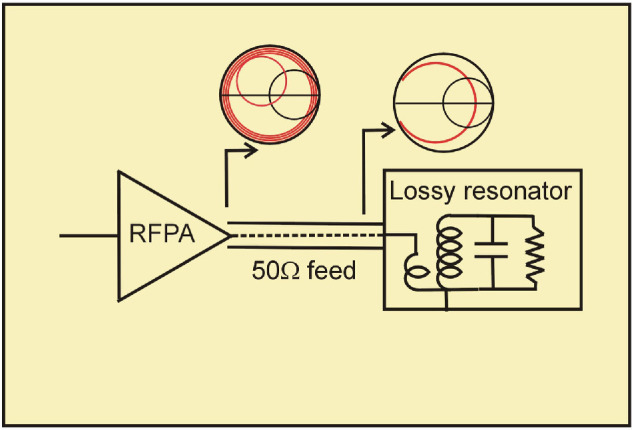
Typical ISM system.

But a typical ISM applicator presents a highly variable load to the PA, often taking the characteristics of a lossy resonator (figure 2).

**Figure 3.**
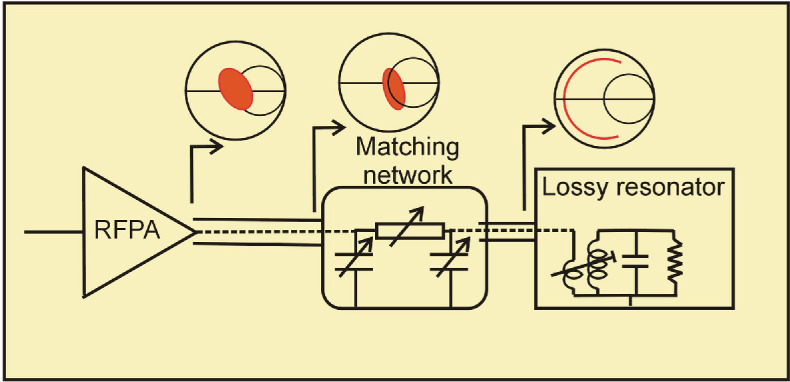
Target impedance matched to 50 Ohms.

The impedance presented to the RFPA is now highly dependent on the length of the connecting cable, as indicated by the Smith chart insets. The conventional approach to relieving this problem is to design a matching network to convert the target device impedance to 50 Ohms, as shown in figure 3.

Radio frequency (RF) matching theory shows that at least two reactive elements, usually implemented as capacitors, are required to achieve this matched condition. So a typical ISM application will require the ability to vary these tuning elements as the load impedance varies, both from sample to sample and possibly interactively as the sample heats up. Rather than attempting to use variable reactance elements such as varactors, which are lossy and cannot withstand high voltages, in an ISM application, it is conceivable to use frequency as an adjustable parameter.

**Figure 4.**
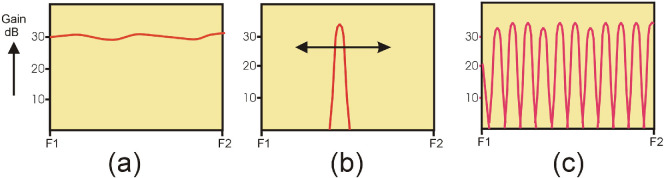
(a) Conventional PA, broadband response, (b) tuner approach, (c) ‘swept match’ approach using frequency as an independent variable.

The tuner approach, shown in figure 4*b* requires the use of interactive adjustable elements; in an ISM application, these can conceivably be mechanically controlled, but electromagnetic drivers and extensive software controls are required. Figure 4*c* indicates a different approach altogether for RFPA design, whereby frequency is not specified and can be used as an independent variable. This can be implemented using a long phase length element and a single variable tuning element.

## 3. Pulsed microwave sources

ISM applications, other than those requiring raw heating, do not necessarily require a continuous supply of RF power; non-thermal biological exposure such as in electroporation can be successful using short pulses.

This is a controversial area. The basic reason for the use of the term non-thermal is that if microwave power of *P* watts is applied in short pulses of duration *τ* at a repetition time period of *T*, the mean power being supplied to the test sample is *Pτ*/*T*, where *τ*/*T* is termed the ‘duty cycle’ and usually expressed as a percentage. So when the duty cycle is 1%, and *P* is 100 W, the mean power supplied would be 1 W, which for a large material sample would not increase the temperature significantly. And even for smaller samples, the pulse length can be reduced almost indefinitely down to the nanosecond level, where effects have still been observed. This, however, does not exclude the possibility of localized heating effects [1]. The controversy, however, focuses on the question: If not heating, what is the cause of the observed changes? It is a well-worn argument, for example, that at an atomic level a microwave ‘wave’ has a quantum energy many orders of magnitude lower than that which could cause an electron to change its orbit, thus manifesting a chemical change. Cells, however, are assemblages of perhaps 10^10^ atoms, and can start to display behavioural responses that are bulk, rather than atomic, level in origin. For example, if nonlinear conduction effects are present, which is quite likely in biological tissue, the microwave signal will be rectified and a direct-current component generated, which could cause behavioural changes in the cell. In simple terms, cells could experience an electric shock. In any event, there is a need for microwave sources that generate such pulsed outputs.

To date, many of these results have been obtained using commercial RFPAs, which are specified for continuous operation. Although this may be satisfactory for research purposes, it appears that some ISM applications will only require a potentially lower-cost source of pulsed microwave power. It is logical to assume that the lower mean power of a pulsed source would use correspondingly lower power, and hence cheaper, transistors. This, however, is not usually the case.

**Figure 5.**
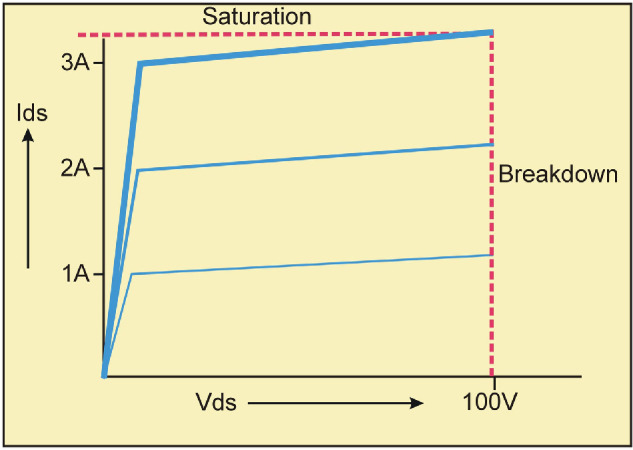
RF power transistor; both voltage and current have well-defined ‘safe’ limits.

**Figure 6.**
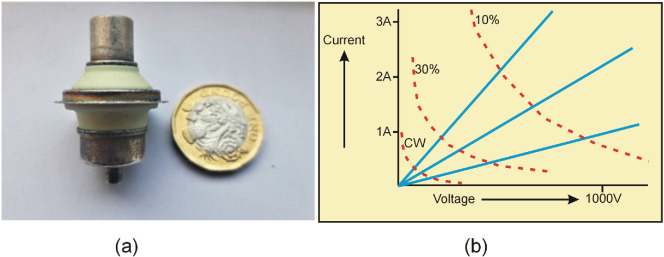
Modern vacuum triode and typical characteristics.

As illustrated in figure 5, a typical microwave power transistor cannot deliver significantly more power in short pulse, low duty-cycle operation than in continuous operation, due to the saturation limit on current and the breakdown limit on voltage. Ironically, it is a largely obsolete device, which has ideal characteristics for exploiting low duty-cycle pulsed operation: the vacuum triode, illustrated in figure 6.

Such devices are still manufactured in some parts of the world and show considerable microwave performance improvement from their Western ancestors that were largely discontinued by the 1970s; in particular, they display useful gain up to at least 3 GHz. They are much more compact than a magnetron (as shown in figure 6*a*), have less demanding power supply requirements and can be tuned over a wide frequency range. Figure 6*b* shows a typical set of direct-current characteristics that show that the vacuum device has much reduced constraints on the current and voltage, and operation is almost entirely constrained by thermal considerations. Consequently, the operating power level can be increased as the pulse duty cycle is decreased, as indicated in figure 6*b*.

## 4. The forgotten TRAPATT

There is another forgotten technology that may be more worth consideration for pulsed applications: the avalanche diode oscillator, and in particular the trapped plasma and avalanche triggered transit (TRAPATT). This device, which is a simple diode structure, was extensively researched in the pre-gallium arsenide era [2–4] and was demonstrated to be able to generate hundreds of watts at frequencies up to several GHz for short pulse durations.

**Figure 7.**
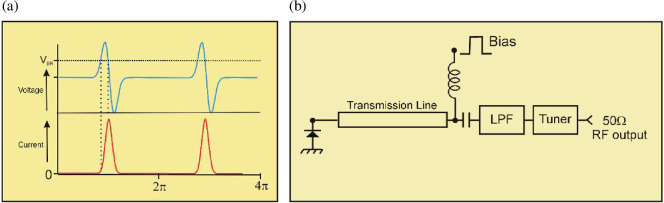
(a) Avalanche diode (TRAPATT) waveforms. (b) TRAPATT oscillator test circuit.

As shown in figure 7*a*, the diode is swept into avalanche breakdown and the current rapidly multiplies up to a peak value of many amps before the action of the passive circuit causes the voltage to collapse and the cycle repeats. These diodes were simple structures, and given that this research was conducted in the late 1960s to early 1970s era, it is tempting to speculate on what could be achieved using modern semiconductor manufacturing technology, especially in the area of heat removal that was essentially the Achilles heel of the TRAPATT. If the ISM application zone really requires a cheap, compact, pulsed microwave source the TRAPATT could well be worth at least a revisit.

In pursuit of this possibility, it has been possible to revive some work that was published in the early 1970s by the current author and others [3–6], which showed, *inter alia*, that TRAPATT oscillations could be observed using cheap computer switching diodes. The motivation for this effort can be summarized as follows:

— Re-acquaint the potential user communities with simpler and lower-cost alternatives to generating GHz frequency microwave power than the current widespread use of expensive compound semiconductor transistor technologies such as gallium nitride (GaN), not to mention the cumbersome and frequency-limited magnetron inside every domestic microwave oven.
— Through practical demonstration, confirm that the TRAPATT mode is still viable, using modern instrumentation, which was not available in the 1970s era.
— Re-address the possibilities offered by half a century of progress in semiconductor technology and manufacturing techniques. In particular, it should be possible to solve two of the detrimental aspects of TRAPATT oscillators, namely, a slow build-up of oscillation from initial bias application and the need for vastly improved heat sinking to handle the high power density.

Figure 7*b* shows the block diagram of a TRAPATT test circuit. It consists essentially of an approximate half-wavelength transmission line which is terminated with a low-pass filter the cut-off frequency of which is designed to pass the fundamental frequency but present a short circuit at higher harmonics. This is followed by a separate matching section, which for these measurements was implemented with an external microwave twin slug tuner.

**Figure 8.**
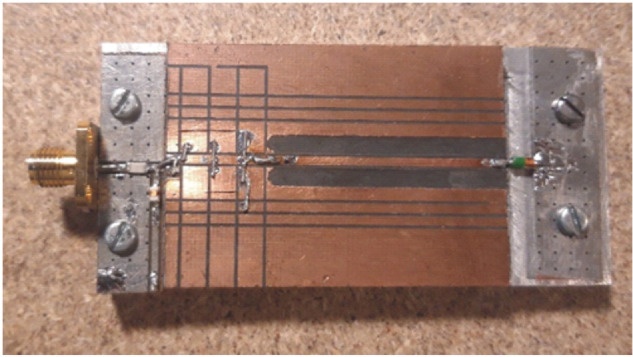
2.4 GHz Microstrip TRAPATT diode test fixture.

Figure 8 shows the implementation of this circuit using a high-frequency printed circuit board. The TRAPATT transmission line is a microstrip line having a characteristic impedance of 70 Ohms and the low-pass filter is a ‘pi’ section consisting of open-circuit microstrip stubs. The high-voltage pulsed bias is introduced through a surface mount inductor of approximately 20 nH.

A number of different diode types have been tested in this circuit, with varying results. Breakdown voltage, junction capacitance, package inductance and the basic doping structure all play a part in determining the interaction between the dynamic diode breakdown characteristic and the passive circuit impedances at several harmonic frequencies. This interaction process was never fully resolved in the numerous papers that appeared in the literature in the late 1960s to early 1970s era; commercial computer circuit analysis software, and even computer access, was not widely available at the time. As such, for an initial revisit to the subject, some older diode types that were cited in the literature at the time have been sourced. For example, the 1S44 is a typical high-speed wire-ended, glass-packaged switching diode, having a capacitance of 1−2 pF and a static voltage breakdown in the 100−150 V range. Although now a legacy item, similar diodes are still manufactured in large quantities today, and ubiquitous types such as the 1N914 are quite capable of TRAPATT oscillation at lower GHz frequencies.

A critical aspect of TRAPATT oscillation is the very high heat dissipation, which will vastly exceed the specification of a small wire-ended package. Given that the typical bias supply would be over 100 V, at 1−2 A, bias pulse lengths of well under 1 µs need be employed using these packages, but it should be noted that such short pulse lengths are not a fundamental limitation of the oscillation itself.

**Figure 9.**
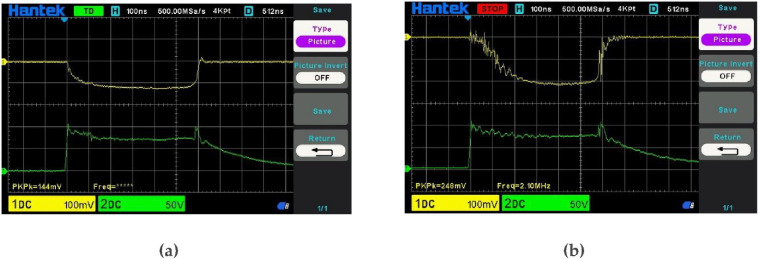
TRAPATT bias waveforms; (a) voltage (lower, 50 V/div), current (upper, 1 A/div); (b) detected RF output (upper, 10 W/div).

Figure 9*a* shows a typical TRAPATT response in the bias time domain. The bias voltage across the diode drops below the breakdown value of 105 V as TRAPATT oscillation builds up over a time period of a few hundred nanoseconds. Figure 9b shows the detected RF output at 2.4 GHz, showing in this case a power of approximately 20 W.

**Figure 10.**
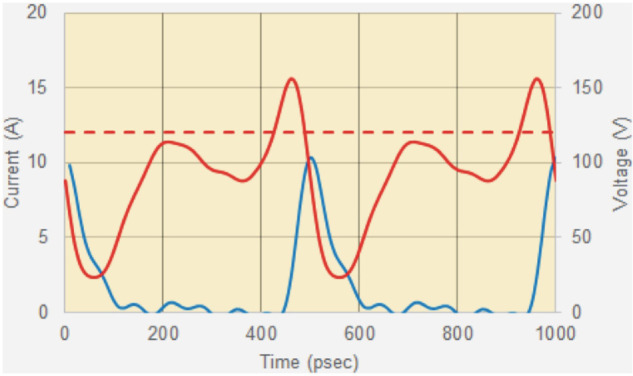
1S44 TRAPATT RF waveforms: voltage (red, r/h scale, volts, breakdown level shown dotted), current (blue, l/h scale, amps); see text for comments on probe calibration.

Figure 10 shows the corresponding RF time domain waveforms that can be sampled using suitable broadband probes. Such probes pose difficult design and calibration issues and should be regarded as displaying qualitative, rather than accurately quantitative [7] waveforms . These plots do, however, give a useful indication of the large signal oscillation properties. Note how the voltage swings well above the breakdown level, then collapses very quickly within 50 picoseconds as the avalanche current escalates, as predicted in the theoretical plots shown in figure 7. The avalanche current starts to rise as the voltage passes through the breakdown level and continues to multiply until the voltage drops back down below breakdown so that the current peak coincides with the point where the voltage drops below the breakdown level.

These results represent what can reasonably be claimed to be the first observation of high-power TRAPATT oscillation for several decades and serve as a ‘reality check’ for the existence of this now largely forgotten means of high-power microwave generation. Power levels of 20 W are, of course, now readily available from commercial amplifiers using GaN transistors, but these devices are expensive items (£100 per transistor at this power level), and the striking divergence in cost and circuit complexity will increase at higher power levels. This is merely a viability demonstration of a potentially useful source of microwave power that could easily, and has historically, delivered hundreds of watts of pulsed power. Its reincarnation using modern semiconductor technology and fabrication techniques is thus a tantalizing prospect.

## 5. Conclusions

ISM RFPA requirements are sufficiently different from the more conventional telecom and military applications that some fundamental changes in design philosophy are necessary. Non-thermal effects in biological and medical applications are being observed and could lead to commercial products that require a substantially different approach to RFPA design and technology. In particular, the observations that important changes in biological cells can be engineered using short pulses of microwave radiation may create a new commercial requirement for cheap and compact microwave sources.

Currently available high-power microwave sources are based around transistors using expensive semiconductor processes such as GaN, which are more suited to generating continuous, rather than pulsed, power. Microwave semiconductor research has, over many decades, sought a ‘holy grail’ of a device technology that displays higher transconductance per unit of current. To date, this research has pursued two paths: exotic higher mobility materials and the fabrication of sub-micron physical features. Avalanche breakdown provides an alternative path which to date has been little exploited, in part due to the heat management problem and the inherent noise associated with the avalanche process. A device such as the TRAPATT could benefit from a revival, and using modern semiconductor technology could provide a cheap and compact source of pulsed microwaves at GHz frequencies. It should also be noted that solutions to the fundamental limitations imposed by a two-terminal device were also explored and demonstrated in this era [8]. ISM applications would appear to offer an application space where these limitations could be manageable.

## Data Availability

This article has no additional data.
